# Development of the membrane ceiling method for in vitro spermatogenesis

**DOI:** 10.1038/s41598-024-84965-1

**Published:** 2025-01-03

**Authors:** Maki Kamoshita, Hiroki Shirai, Hiroko Nakamura, Tetsuya Kishimoto, Yuki Hatanaka, Daisuke Mashiko, Katsuhiro Esashika, Jingjing Yang, Satoshi Yamasaki, Takehiko Ogawa, Hiroshi Kimura, Masahito Ikawa

**Affiliations:** 1 Research Institute for Microbial Diseases, Osaka University, Osaka, Japan; 2 Micro/Nano Technology Center, Tokai University, Kanagawa, Japan; 3 Immunology Frontier Research Center, Osaka University, Osaka, Japan; 4 Biotechnology Department, Synthetic Chemicals Laboratory, R&D Center, Mitsui Chemicals, Inc, Chiba, Japan; 5 Marketing & Innovation Department, New Business Incubation Center, Mitsui Chemicals, Inc, Tokyo, Japan; 6 Institute of Molecular Medicine and Life Science, Yokohama City University, Kanagawa, Japan; 7 The Institute of Medical Science, The University of Tokyo, Tokyo, Japan; 8 Center for Infectious Disease Education and Research, Osaka University, Osaka, Japan

**Keywords:** In vitro spermatogenesis, Testis culture, Polydimethylsiloxane (PDMS), 4-Polymethyl-1-pentene polymer (PMP), Fluorinated ethylene-propylene copolymer (FEP), Membrane ceiling chip, Germline development, Biomedical engineering

## Abstract

Spermatogenesis is one of the most complex processes of cell differentiation and its failure is a major cause of male infertility. Therefore, a proper model that recapitulates spermatogenesis in vitro has been long sought out for basic and clinical research. Testis organ culture using the gas-liquid interphase method has been shown to support spermatogenesis in mice and rats. However, the conventional method using agarose gel has limitations including medium replacement efficiency and live imaging because agarose absorbs medium and is not transparent. To overcome this issue, we developed a new device using microporous membranes and oxygen-permeable materials. Mouse testes sandwiched between a microporous polyethylene terephthalate (PET) membrane on top and an oxygen-permeable 4-polymethyl-1-pentene polymer (PMP) membrane base maintained spermatogenesis over months. The chamber volume was minimized to 0.1% of the culture medium. Weekly time-lapse live imaging enabled us to observe transgenically fluorescent acrosome and nuclear shape formation throughout spermatogenesis. Finally, we obtained healthy fertile offspring from spermatozoa generated in our system. The device could be used not only for basic research to understand spermatogenesis but also for applied research, such as diagnosing and treating male infertility.

**Supplementary Information:**

The online version contains supplementary material available at 10.1038/s41598-024-84965-1.

## Introduction

Approximately 70–90% of male infertility is attributed to spermatogenesis defects^1^. Spermatogenesis occurs in the seminiferous tubule in the testis and multiple steps proceed spatiotemporally, including spermatogonial cell proliferation, meiosis, and spermiogenesis. The process is tightly regulated by testicular somatic cells such as the Sertoli cells, Leydig cells, and peritubular myoid cells^2^, and over 1000 genes are testis-enriched genes^3^. Due to its complexity and the lack of a proper in vitro system, spermatogenesis has been mainly studied in animal models, such as drug-administrated or gene-manipulated mice. However, conventional histological analysis requires sacrificing the animal model at each time point, making it difficult to research spermatogenesis in a single individual chronologically.

Recent advances in live imaging technologies have been challenging this issue. The combination of transgenic mice expressing GFP-tagged proteins in germ cells with low-invasive and high-resolution fluorescent microscopes enables researchers to observe spermatogenesis in live animals under anesthesia for 4 days^4,5^. However, the anesthesia prevented the mice from eating and drinking, which raises concerns regarding its use from an animal welfare standpoint. It is important to note that spermatogenesis in mice lasts approximately 35 days^2^ so a 4-day period is insufficient to observe the complete process. Thus, an effective system to replicate spermatogenesis in vitro is necessary.

In 2011, Sato et al. succeeded in cultivating mouse testis and generating sperm in vitro^6^. They divided the neonatal testis into small pieces, placed them on agarose (AG) in a well plate, and added medium up to half of the AG height (the AG method). This “gas-liquid interphase” culture, supplying air from the top and nutrients from the bottom, is critical for the success of in vitro spermatogenesis. However, when the tissue is cultured in a dome shape on the AG, the center becomes necrotic due to insufficient oxygen and nutrient supply. Therefore, they improved the AG method by placing a gas-permeable PDMS (polydimethylsiloxane) ceiling chip (PC chip) over the tissue to flatten it evenly^7^, (the AG-PC method). Even so, thick AG (about 5 mm) prevents observation from the bottom, and the risk of contamination by opening the plate cap prevents long-term observation from the top^8^.

In vitro spermatogenesis systems are also important from an animal welfare perspective. For example, one can prepare more than 20 tissue pieces (about 1–2 mm × 1–2 mm × 0.1 mm per piece) from a single neonatal mouse at seven days postpartum (see “Testis tissue culture” section in “Materials and methods”), and examine them under different conditions, while one mouse is generally treated with one condition in vivo. Future applications using ESC/iPSC-derived organoids may lead to animal-free spermatogenesis research^8^.

In this study, we developed a novel device, the membrane-ceiling (MC) chip, comprised of a microporous membrane and polyimide double-sided tape positioned on a plate made from oxygen-permeable material (the MC method). We demonstrated its application for long-term testicular tissue culture and time-lapse live imaging analysis.

## Result and discussion

### Generation of GFP-tagged acrosomes and RFP-tagged nuclei (GARN) mice for monitoring spermatogenesis in vitro

Acr-Acr3-GFP (Acr-GFP) and Prm1-H3.3-mCherry (H3.3-mCherry) transgenic (Tg) mice have been useful in monitoring spermatogenesis in vitro. Spermatogenic cells exhibit green fluorescence starting from the pachytene spermatocyte stage, with Acr-GFP labeling the acrosome as it transitions from bowl-shaped to crescent-shaped, and red fluorescence starting from step 11 spermatid to spermatozoa, with H3.3-mCherry labeling the nucleus^9^. In the present study, we generated a double Tg mouse line (GARN; GFP-tagged Acrosomes and RFP-tagged Nuclei) carrying Acr-Acr3-GFP and Prm1-H3.3-mCherry transgenes (Supplementary Fig. S1). The GARN Tg male mice are fully fertile.

### MC method and device design

**Fig. 1:**
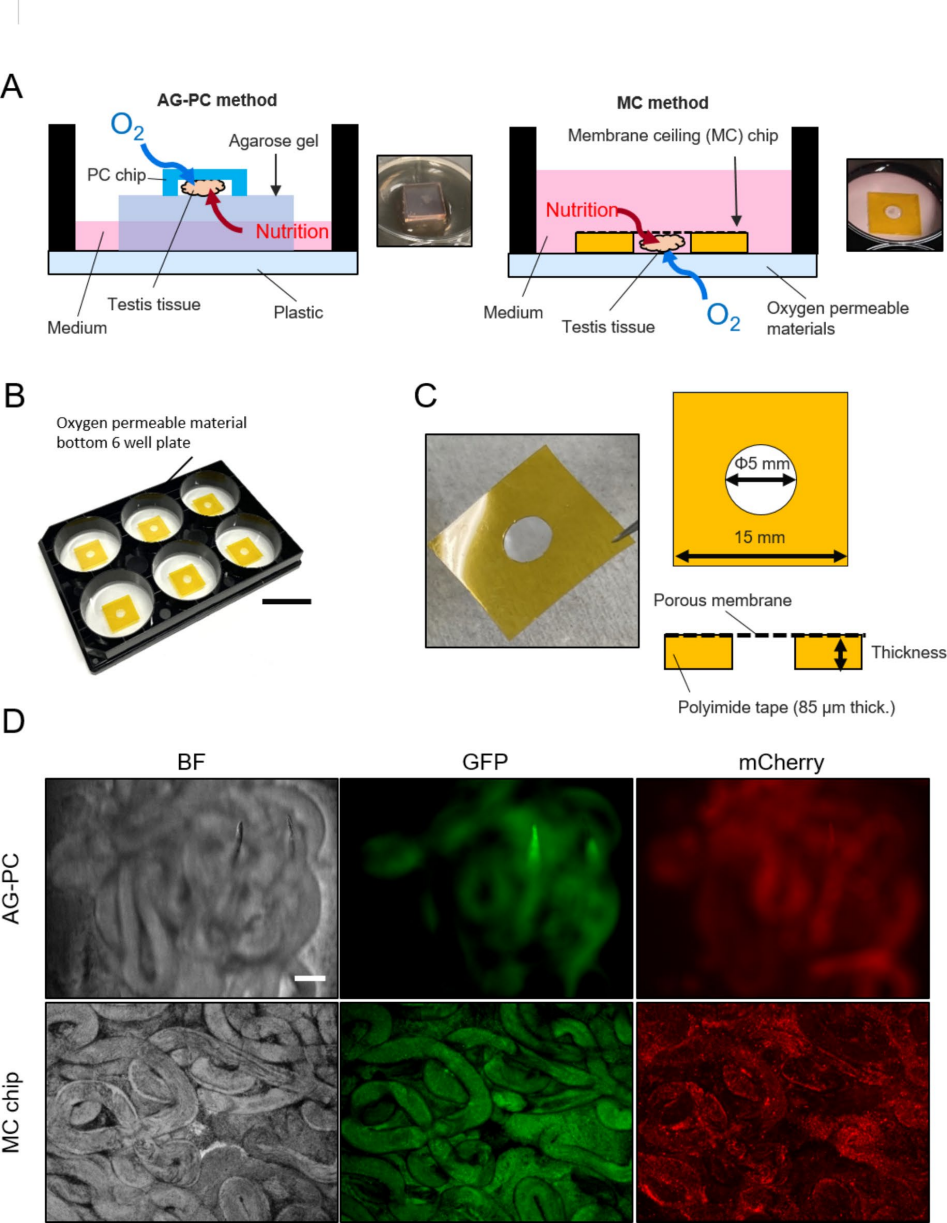
Device design. (**A**) Schematic illustrations and pictures of the conventional method using agarose gel and PDMS ceiling chip (AG-PC) (left) and our novel method consisting of the membrane ceiling (MC) chip and an oxygen-permeable material (MC method) (right). (**B**) Picture of the MC method. Scale bar = 25 mm. (**C**) Picture and schematic illustrations of the MC chip. (**D**) Representative images of testis tissue in the AG-PC and MC method observed by an inverted microscope (BZ-X700). PDMS was used to line the base of the well plate. Scale bar = 100 μm.

To culture tissues in the gas-liquid interfacial phase without agarose gel, we designed a device consisting of a microporous membrane with double-sided tape (MC chip) and gas-permeable material (the MC method, Fig. 1A,B)^10^. When we sandwich the tissue, nutrients are supplied through the upper microporous membrane, and oxygen is supplied through the lower gas-permeable material (Fig. 1A). We can adjust the distance between the microporous membrane and the gas-permeable material by changing the thickness of a double-sided tape (Fig. 1C). Notably, our device improved the medium exchange rate. In the AG method, the medium can not be replaced efficiently because the agarose block (10 mm × 10 mm × 5 mm = 500 mm^3^) is approximately 25% of 2 mL of culture medium^11^, while the tissue chamber volume is reduced to approximately 0.1% of 2 mL of culture medium in the MC method (2.5 mm × 2.5 mm × 3.14  × 0.085 mm = 1.7 mm^3^) (Fig. 1C).

When we observed the testis tissues collected from adult GARN mice, we had clearer images using the MC method compared to the AG-PC method (Fig. 1D). We confirmed that testis tissues in the MC method could be observed by inverted microscopes with time-lapse and tiling function (Biostation CT and BZ-X700) (see “Microscopy” section in “Materials and methods”).

### Optimization of microporous membranes for MC chip

**Fig. 2:**
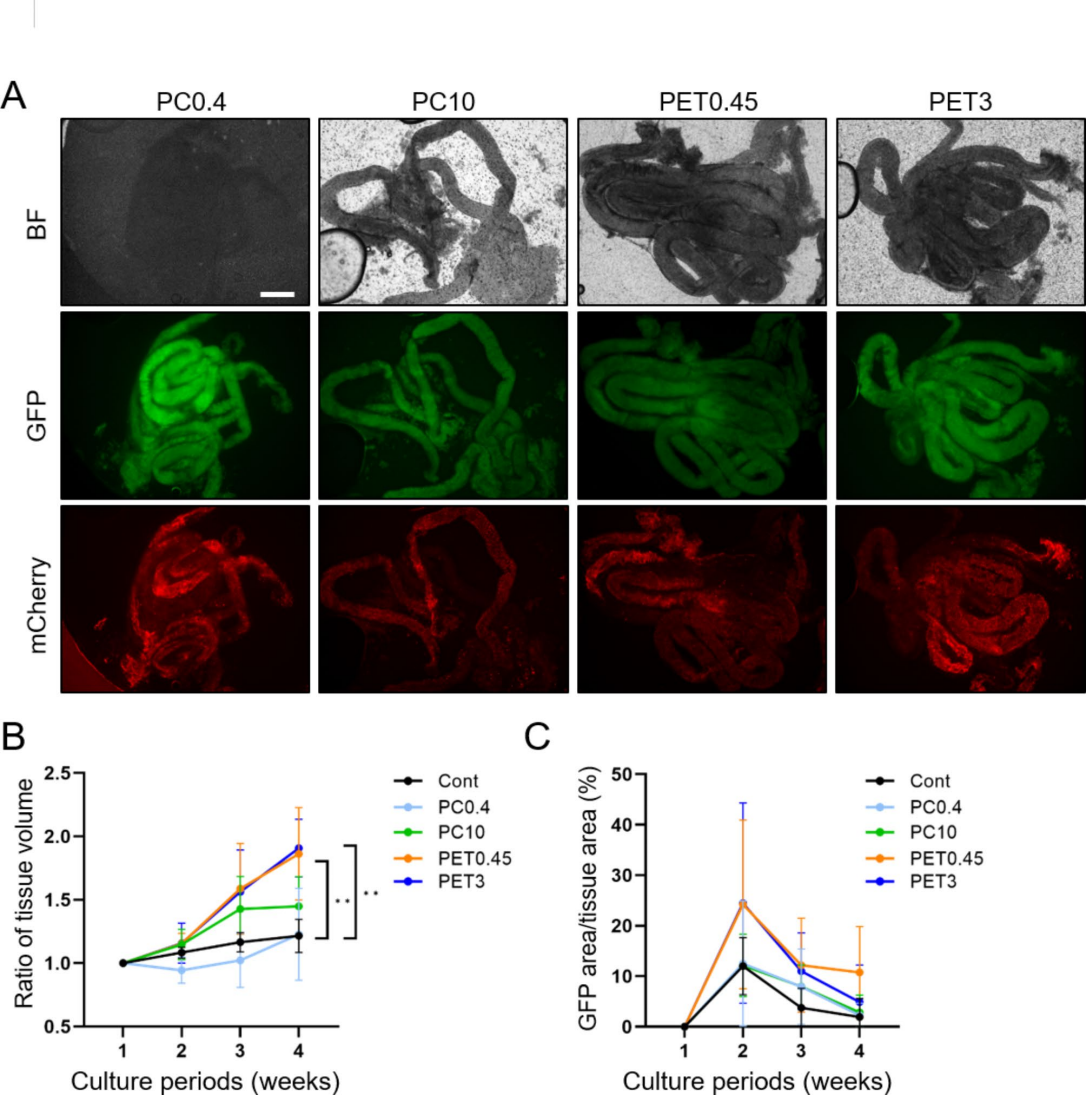
Optimization of porous membranes used for the MC chip. (**A**) Representative images using an inverted microscope (BZ-X700) of the MC chip made of various porous membranes as indicated. Scale bar = 0.5 mm. (**B**) The ratio of tissue volume expansion and (**C**) the GFP-positive rate over 4 weeks with MC chips consisting of indicated membranes. **P < 0.01 and *P* > 0.05 if no indication. Twelve (Cont), 8 (PC0.4), 8 (PC10), 8 (PET0.45), and 8 (PET3) tissues were used for the experiments. Images were taken by IX73 for quantifications.

As indicated in Supplementary Table S1, testis tissues were cultured using the MC chip consisting of various microporous membranes, polyethylene terephthalate (PET) membrane with 0.45 μm φ pore (PET-0.45) or 3 μm φ pore (PET-3), and polycarbonate (PC) membrane with 0.4 μm φ pore (PC-0.4) or 10 μm φ pore (PC-10). The PET-0.45 provided better bright field images than the other membranes (Fig. 2A). When we cultivated 7-day postpartum testis tissues for 4 weeks, tissues expanded approximately twice as wide as the original with PET membranes while the tissues expanded less than 1.5 times with the other membranes or AG-PC control (Fig. 2B). Because GFP positive spermatocytes appear around two weeks of age in vivo, we measured the GFP-positive area rate (GFP-positive area/total tissue area) as an indication for early spermatogenesis. The rate reached its peak at the 2-week mark and then decreased similar to the AG-PC control. The PET membranes gave a relatively better GFP-positive area rate but there were no significant differences among the membranes (Fig. 2C). From these results, we used PET-0.45 to generate the MC chip for further experiments.

### Optimization of oxygen-permeable materials

**Fig. 3:**
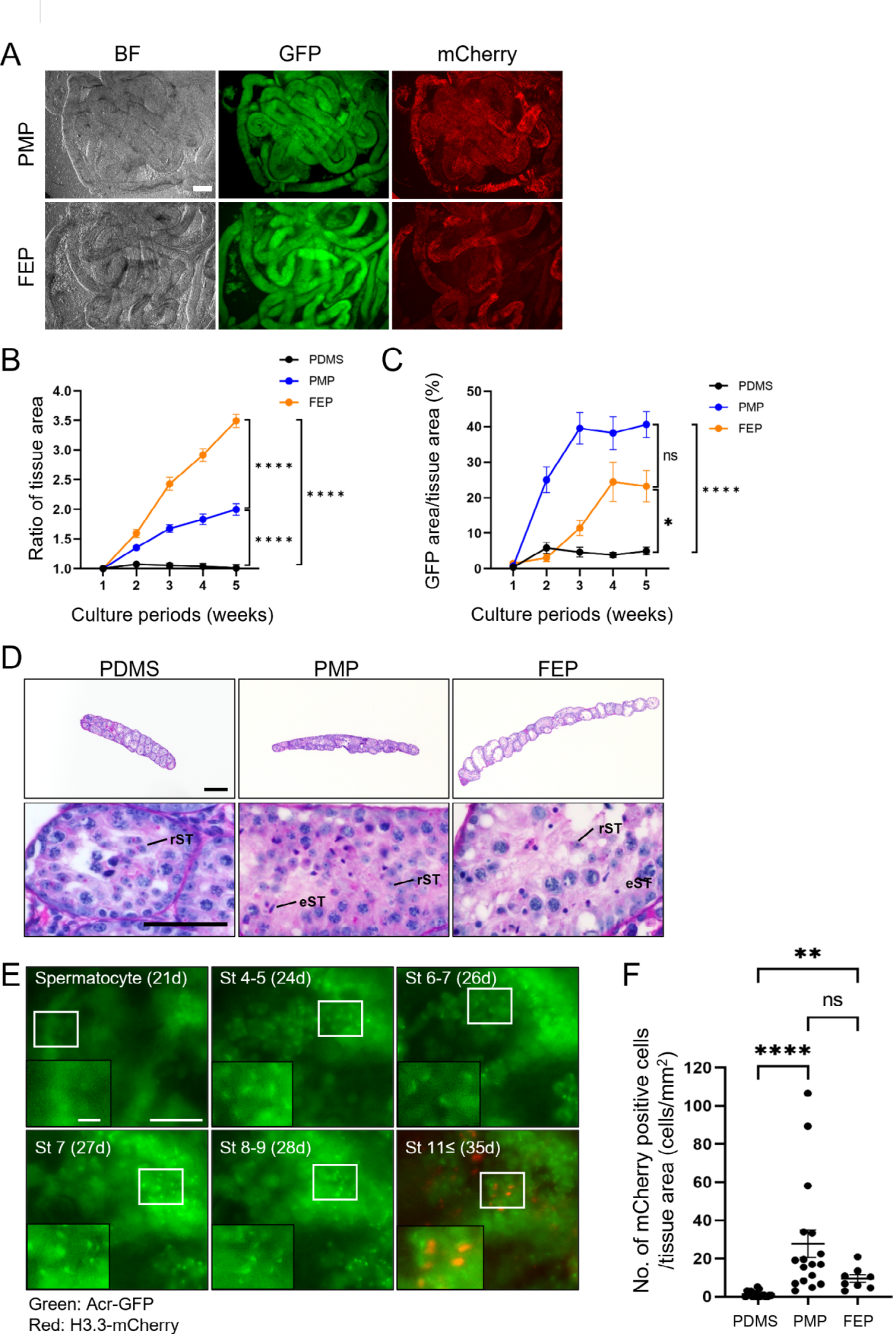
Optimization of oxygen-permeable materials for device base. (**A**) Representative images showing the visibility of seminiferous tubules collected from GARN adult mice in the device using PMP or FEP. Images were taken by an inverted microscope (BZ-X700). Scale bar = 100 μm. (**B**) The ratio of tissue area expansion and (**C**) the GFP-positive rate for 5 weeks in the device with PDMS, PMP, or FEP base plate or dish. *P < 0.05, ****P < 0.0001, and ns; no significant. (**D**) Representative images of He-PAS staining of testis sections after culturing in the device with PDMS, PMP, or FEP base plate or dish. *rST* round spermatids, *eST* elongating spermatids. Scale bar = 200 μm (upper panel) and 50 μm (lower panel). (**E**) Live imaging of in vitro spermatogenesis of the same tubule during cultivation on the PMP bottom plate. The images were taken by BioStation CT. *St* step of spermatids. *d* culture days. Scale bar = 50 μm and 10 μm (zoomed in). (**F**) The number of mCherry-positive cells divided by tissue area. Each dot shows the average cell number per tissue area of frames. Images were taken at 5 weeks in the device with PDMS, PMP, and FEP using the tiling function of a microscope (BioStation CT). **P < 0.01, ****P < 0.0001, and ns; no significant. Seventeen (PDMS), 17 (PMP), 8 (FEP) tissues were used for quantification.

According to previous studies^9,12,13^, oxygen is an important factor in regulating spermatogenesis. However, cultures with different oxygen concentrations require several incubators and it is difficult to maintain lower oxygen concentrations during observation outside the incubators. Thus, we tried to regulate oxygen levels in the tissue (oxygen tension), by testing different gas-permeable materials at the base. Because PDMS, PMP, and fluorinated ethylene-propylene copolymer (FEP) have different oxygen fluxes (Supplementary Table S2), we cultivated 7-day postpartum GARN testis tissues using the MC method with PDMS, PMP, and FEP for 5 weeks. We first confirmed that PMP and FEP didn’t affect the visibility of seminiferous tubules by bright field and GFP/mCherry fluorescences (Fig. 3A). The total transmittance of PMP and FEP is comparable to glass, and PMP exhibits particularly low autofluorescence^14–16^.

The tissue expanded most with FEP among the tested materials (Fig. 3B), however, the GFP-positive area rate was highest with PMP (PDMS = 5.0 ± 1.1%, PMP = 40.6 ± 3.6%, and FEP = 23.2 ± 4.4%) (Fig. 3C and Supplementary Fig. S2A). Consistent with this observation, He-PAS staining at 5 weeks culture with FEP resulted in expanded tubules with fewer cells in them (Fig. 3D, upper panels). More importantly, we found more elongating spermatids in tissues cultivated with PMP and FEP (Fig. 3D, lower panels). When we calculated the number of mCherry-positive cells (step 11 or later haploid cells) (Fig. 3E) and normalized them by tissue area, the scores were higher with PMP (27.8 ± 7.2 cells/mm^2^) and FEP (9.5 ± 2.0 cells/mm^2^) than PDMS (1.3 ± 1.3 cells/mm^2^) (Fig. 3F). The score was higher with PMP than FEP but we did not see any significant differences (*P* = 0.06316).

We then calculated the theoretical oxygen flux to understand the optimal oxygen supply rate for mouse testis culture. In our culture device using the MC chip, oxygen is supplied not only from the base material but also from the medium; thus, the oxygen supply rate (*OSR*) was determined by summing the oxygen fluxes on the oxygen-permeable material (*F*_material_) and the medium (*F*_medium_)^17^:

1 $$\:OSR={F}_{\text{m}\text{a}\text{t}\text{e}\text{r}\text{i}\text{a}\text{l}}+{F}_{\text{m}\text{e}\text{d}\text{i}\text{u}\text{m}}$$

The theoretical oxygen flux of the oxygen-permeable material and the medium can be calculated by using Fick’s law^18^:

2 $$\:{F}_{\text{m}\text{a}\text{t}\text{e}\text{r}\text{i}\text{a}\text{l}}={P}_{\text{m}\text{a}\text{t}\text{e}\text{r}\text{i}\text{a}\text{l}}\times\:\frac{({p}_{\text{a}\text{i}\text{r}}-{p}_{\text{t}\text{e}\text{s}\text{t}\text{i}\text{s}})}{{z}_{material}}$$

3 $$\:{F}_{\text{m}\text{e}\text{d}\text{i}\text{u}\text{m}}={P}_{\text{m}\text{e}\text{d}\text{i}\text{u}\text{m}}\times\:\frac{({p}_{\text{a}\text{i}\text{r}}-{p}_{\text{t}\text{e}\text{s}\text{t}\text{i}\text{s}})}{{z}_{medium}}$$

where *P* is oxygen permeability, *p* is oxygen partial pressure, and *z* is thickness. By assuming that the partial pressure in the testis (*p*_testis_) is zero, each theoretical maximum oxygen flux can be obtained (Supplementary Fig. S2B). Values from previously published literature were used for each parameter as shown in Supplementary Table S2. The results of the calculation showed that the maximum *OSR* was PDMS > PMP > FEP. Based on these results, poor development of testis tissues using PDMS might have been due to oxidative stress on the tissue caused by the high oxygen supply rate of PDMS. In cultures using FEP, although the growth rate of testis tissue was high, the low cell density and Acr-GFP expression rate might have been due to hypoxia causing germ cells to die, allowing surviving stromal cells to proliferate.

As described above, lower oxygen concentrations (10 and 15%) in incubators improved in vitro spermatogenesis efficiency in mouse testis^9,13^. Interestingly, the maximum *OSR* in PMP (*z* = 50 μm) (376.1 pmol/cm^2^ s) was similar to the AG-PC method with 10% (312.8 pmol/cm^2^ s) and 15% (468.8 pmol/cm^2^ s) oxygen, suggesting about 313–469 pmol/cm^2^ s *OSR* is optimal oxygen condition for spermatogenesis in vitro. Taken together, we concluded that using PMP (*z* = 50 μm) as the oxygen-permeable material in testis tissue culture with the MC chip enables efficient spermatogenesis in vitro in the air oxygen condition. According to Hashimoto et al.^9^ the oxygen gradient between the sides of the basal membrane and lumen in seminiferous tubules in vivo may exist and this gradient is important for appropriate spermatogenesis. Therefore, the development of a device that can control oxygen levels spatiotemporally is required to further reproduce spermatogenesis in vitro.

With the MC method, we succeeded in monitoring spermatogenesis by time-lapse imaging of the entire tissue (Supplementary movies S1, and S2) and focusing on the same point of the tube (Fig. 3E and Supplementary movies S3). We also succeeded in observing organelle formation with 2D (Supplementary Fig. S3A) and 3D (Supplementary Fig. S3B and Supplementary Movie S4) using a confocal microscope. This is advantageous over the AG-PC method, where cultured testis tissues needed to be transferred onto a slide glass to observe in high resolution^11^.

### Culture medium for the MC method

**Fig. 4:**
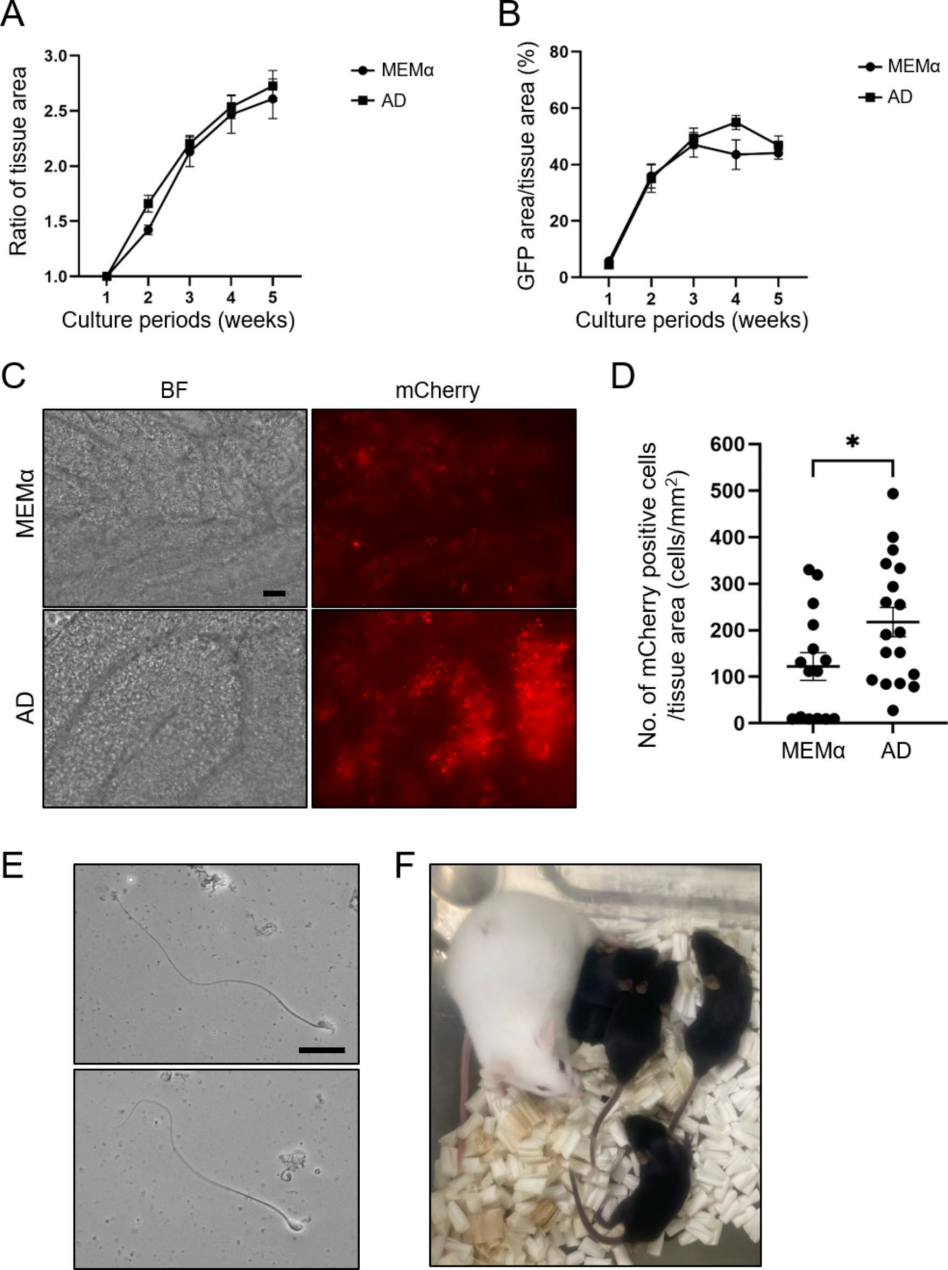
The effect of advanced DMEM/F12 (AD) as the in vitro basal medium in spermatogenesis. (**A**) The ratio of tissue area expansion and (**B**) the GFP-positive rate for 5 weeks in MEMα or AD-based culture medium. There were no significant differences (*P* > 0.05). (**C**) Representative images of mCherry-positive cells observed using an inverted microscope (BZ-X700) after 5 weeks cultivated in MEMα or AD-based culture medium. Scale bar = 50 μm. (**D**) The number of mCherry-positive cells divided by tissue area (mm^2^). Each dot shows the average cell number per tissue area of frames. Images were taken at 5 weeks using the tiling function of a microscope (BioStation CT and BZ-X700). *P < 0.05. Fifteen (MEMα) and 18 (AD) tissues were used for quantification. (**E**) In vitro generated sperm after 5 weeks in AD-based culture medium. The images were taken using an upright microscope (BX53). Flagellated cells with normal (upper panel) or abnormal (lower panel) heads were used for ICSI. Scale bar = 20 μm. (**F**) A picture of obtained offspring by ICSI using in vitro generated sperm.

We examined different mediums with our new device to further improve in vitro spermatogenesis efficiency. MEMα was reported to be better than DMEM and StemPro-34 as a base medium for in vitro spermatogenesis with the AG method^19^. In a previous study, a germline stem cell medium was used for seminiferous tubule culture, but Sertoli cells grew rather than germ cells^7^, suggesting that the development of a proper medium for both somatic cells and germ cells is important. Among commercially available media, we found Advanced DMEM/F12 (AD) contains several important factors for spermatogenesis not present in MEMα, such as insulin^7^, transferrin^20^, glutathione^11^, manganese^21^, and selenium^22^. Therefore, we used AD as our base medium. While there were no significant differences between MEMɑ and AD in area expansion and GFP-positive rates (Fig. 4A,B), the number of mCherry-positive cells in AD (217.6 ± 31.3 cells/mm^2^) was higher than in MEMɑ (122.0 ± 29.7 cells/mm^2^) (Fig. 4C,D).

Finally, we evaluated whether in vitro-generated sperm using AD could produce offspring. After 5 weeks of cultivation in AD with weekly live imaging, flagellated spermatozoa (Fig. 4E) were harvested from the seminiferous tubule and used for intracytoplasmic sperm injection (ICSI). Fifty-one 2-cell embryos derived from 91 zygotes were transferred into two pseudopregnant females, and six offspring were delivered (Fig. 4F), confirming that sperm generated from our device is capable of producing offspring after serial live-cell imaging. In addition, we confirmed that these pups developed normally and sire the next generation.

## Conclusion

In this study, we developed the MC-method as a novel liquid-gas interphase culture system by sandwiching tissues between an MC-chip and gas-permeable material, allowing for long-term culture with time-lapse live imaging which had been difficult to achieve using the conventional AG-PC method. As a proof of concept study, we cultivated testicular tubules from our GARN Tg mice and observed acrosome and nuclear formation. Generated spermatozoa in vitro are capable of producing healthy offspring after ICSI. It cannot be denied that this device has several limitations at present before clinical applications: confirmation of the long-term stability of PET and PMP requires, a large tissue or whole organ culture and perfect mimicry of in vivo spermatogenesis are difficult, and many parameters such as oxygen flux and the size of the device must be modified depending on the species. However, this system could also be used for any tissues and ESC/iPSC-derived organoids that may lead to animal-free research, in vitro spermatogenesis in livestock and endangered species, treatment for patients with spermatogenesis failure, and fertility preservation in cancer patients in the future. Our system provides a versatile platform for both basic and clinical investigations.

## Materials and methods

### Testis culture imaging device

We developed a novel MC device that could supply oxygen and nutrients to testis tissue within the focal distance range of an inverted microscope (Fig. 1A). This testis culture device consists of an MC chip and an oxygen-permeable material base (Fig. 1B). The MC chip contains a culture chamber with a microporous membrane ceiling, constructed using polyimide double-sided tape and a microporous membrane (Fig. 1C). Polyimide double-sided tape with thicknesses of 85 μm (API-214 A; Chukoh Chemical Industries, Ltd., Tokyo, Japan) was used to control tissue thickness. The culture chamber (φ5 mm) was prepared by making a hole (Hin-M200; KOKUYO Co., Ltd., Osaka, Japan) on a 15 mm square polyimide double-sided tape (Fig. 1C). The MC chips were assembled by adhering four types of microporous membranes PC 0.4, PC 10.0, PET 0.45, PET 3 (shown in Supplementary Table S1) to one side of the polyimide double-sided tapes. The material for the base of the oxygen-permeable material plate consisted of three types of polymer sheets with different oxygen permeability: PDMS, PMP, and FEP. The PDMS plate was assembled by attaching a PDMS sheet (122 mm × 80 mm, 0.5 mm thickness, PDMS; Fukoku Bussan Co., Ltd., Tokyo, Japan) to the base of a bottomless 6-well plate (CSCSM0063; CS CRIE Co., Ltd., Kyoto, Japan) using a silicone-based adhesive. For the PMP and FEP base plates, InnoCell™ (T-FP006N-01; Mitsui Chemicals, Inc., Tokyo, Japan) and Lumox^®^ (94.6077.333; SARSTEDT AG & Co. KG, Numbrecht, Germany) were used, respectively.

### Animal and ethics

All animal experiments performed in this study were approved by the Institutional Animal Care and Use Committees of Osaka University (Osaka, Japan) and Tokai University (Hiratsuka, Japan) in compliance with the guidelines and regulations for animal experiments (approval code: H30-01-0; approval date: July 4, 2018 in Osaka University; approval code: 232022; approval date: April 1, 2023 in Tokai University). C57BL/6 N mice and pseudopregnant ICRs were purchased from Japan SLC, Inc. (Shizuoka, Japan). Animals were housed in a temperature-controlled environment with 12 h light cycles and free access to food and water. This study was performed in accordance with ARRIVE guidelines (https://arriveguidelines.org/). Decapitation with sharp scissors was performed on neonatal mice and cervical dislocation on female mice for oocyte collection was performed by experts according to AVMA guidelines. (https://www.avma.org/sites/default/files/2020-02/Guidelines-on-Euthanasia-2020.pdf).

### Generation of GFP-tagged Acrosomes and RFP-tagged nuclei (GARN) mice

To generate GARN mice (Supplementary Fig. S1A), we used the Acr-Acr3-GFP plasmid, which was previously reported^23^, and pPB Prm1-H3.3-mCherry plasmid, which was gifted by Dr. Okada^24^ (Fig. S1B). DNAs were linearized by XbaI and HindIII for Acr-GFP and ApaI and HindIII for H3.3-mCherry. Mouse zygotes were obtained from C57BL/6 N mice via in vitro fertilization. The linearized DNAs were injected into one of the pronuclei of zygotes, and the zygotes were cultivated in KSOM to two-cell stage embryos and then transferred into pseudo-pregnant female ICRs. The offspring that had both Acr-GFP and H3.3-mCherry transgenes were used for mating to generate F1 mice. The GFP and mCherry signals of F1 mice were confirmed and the line that had the brightest signals was used (Supplementary Fig. S1C). The mouse line will be open to the public through RIKEN BioResource Research Center or the Center for Animal Resources and Development (CARD), Kumamoto University.

### Testis tissue culture

A testis was collected from the GARN male at 7 day postpartum and cut into 10 pieces following tunica albuginea removal^8^. For the MC method culture, a piece of testis was placed on the center of a well with 2.5 µL organ culture medium, covered by the MC chip, and then filled with 2 mL organ culture medium. Culture basal medium MEMα (Invitrogen, Carlsbad, CA) or Advanced DMEM/F12 (Invitrogen, Carlsbad, CA) was used. Forty mg/mL AlbuMAX I (ThermoFisherScientific, Waltham, MA) and 1:100 Antibiotic-Antimycotic Mixed Stock Solution (100×) (NACALAI TESQUE, INC., Kyoto, Japan) were supplemented to the culture medium^7,8^. For the control culture, the AG-PC method was performed according to a previous report^7^. In brief, a piece of tissue is put on an agarose block (1 cm x 1 cm x 5 mm) in a well of 6 well plates and covered by the PC-chip. Then, 1 mL organ culture medium was added to a well. The culture was performed at 34 ºC under 5% CO_2_ in air. The medium was changed once a week.

### Hematoxylin and periodic acid-Schiff (He-PAS) staining

He-PAS staining was performed according to a previous report^25^ with some modifications. Cultured tissues were fixed with Bouin’s fixative (Polysciences, Inc., Warrington, PA) and embedded in paraffin. The paraffin embedding samples were sectioned, rehydrated, and treated with 1% periodic acid for 15 min, followed by staining with Schiff’s reagent (Wako, Osaka, Japan) for 20 min. Then, the sections were stained with Mayer’s Hematoxylin solution for 1 min.

### Microscopy

To investigate spermatogenesis efficiency, microscopy analysis was performed according to a previous report^9^ with some modifications. In brief, bright field and GFP images were taken by inverted microscopes with a tiling function (BioStation CT; Nikon, Tokyo, Japan, BZ-X700; Keyence Corporation, Osaka, Japan). The tissue area was measured using ImageJ software to evaluate the tissue expansion ratio. When using MC chips with different thicknesses, we calculated the tissue volume by multiplying the area by the thickness of the MC chip. Then, the expansion ratio was calculated as 1 when cultured for a week. The GFP-expressing area was measured after binarization using ImageJ software, and the percentages of the GFP-positive area were calculated by dividing by the tissue area. Because observation of the tissues in the AG-PC method was difficult using BioStation CT and BZ-X700, IX73 (Olympus Corporation, Tokyo, Japan) was used for quantification of Fig. 2B,C. After culturing for 5 weeks, mCherry images were taken to investigate the later steps of spermatogenesis (step 11 ≤) (Supplementary Fig. S1B,C). To quantify mCherry-positive cells, we counted mCherry-positive cells per a frame of 20 times magnification and then divided by the tissue area. Frames showing out-of-focus cells were excluded. Observation of He-PAS stained samples and in vitro generated sperm was performed using an upright microscope (BX53; Olympus Corporation, Tokyo, Japan). To obtain high-resolution and 3D images, a confocal microscope was used (Eclipse Ti; Nikon, Tokyo, Japan).

### Intracytoplasmic sperm injection (ICSI)

ICSI was performed according to previous studies^26–28^ with some modifications. Cumulus oocyte complexes (COCs) were collected from the oviducts of 8 weeks old C57BL/6 N females superovulated by an i.p. injection of 5 IU of pregnant mare gonadotropin (PMSG; Asuka Pharmaceutical Co., Tokyo, Japan) followed by 5 IU of human chorionic gonadotropin (Gonatropin; Asuka Pharmaceutical Co., Tokyo, Japan) 48 h later. Sixteen hours after the hCG injection, COCs were collected from oviducts and cumulus cells were removed using 330 µg/mL hyaluronidase (Wako, Osaka, Japan). Denuded oocytes were then activated with 2.5 mM strontium for 10 min and used for ICSI. Cultured seminiferous tubules were cut into small pieces using tweezers and sperm with flagellum were collected by fine glass capillary. Sperm heads cut by a piezo pulse were injected into oocytes using a piezo-driven micromanipulator (PrimeTech, Ibaraki, Japan). Two-cell embryos derived from zygotes generated by ICSI were transferred into the oviduct of pseudopregnant ICR females under the anesthesia containing medetomidine, midazolam, and butorphanol^29^. Nineteen days after embryo transfer, offspring were delivered via vaginal birth or Caesarean section.

### Statistical analysis

Statistical analysis was performed by GraphPad Prism version 9.5.1 for Windows (GraphPad Software, Boston, MA). All percentage data were subjected to arcsine transformation before statistical analysis. One-way ANOVA and then Dunnett’s multiple comparisons test were used for the various membrane studies (Fig. 2B,C). For the oxygen-permeable materials, one-way ANOVA and Tukey-Kramer’s test (Fig. 3B), or Kruskal-Wallis test and Dunn’s multiple comparisons test (Fig. 3C,F) were used. A two-tailed Welch’s t-test was performed to assess basal medium (Fig. 4A,B,D). Differences were considered significant at *P* < 0.05 (*), *P* < 0.01 (**), and *P* < 0.0001 (****). Data are shown as mean ± standard errors of the means (SEMs).

## Electronic supplementary material

Below is the link to the electronic supplementary material.

Supplementary Material 1

Supplementary Material 2

Supplementary Material 3

Supplementary Material 4

Supplementary Material 5

## Data Availability

The datasets analyzed during the current study are available from the corresponding author upon reasonable request.
