# Supplementary material for: Development of the membrane ceiling method for in vitro spermatogenesis
Source: Sci Rep. 2025 Jan 3;15:625. doi: 10.1038/s41598-024-84965-1 (PMC11699200; doi:10.1038/s41598-024-84965-1)
Supplement: Supplementary file 5 — Supplementary Material 5 [file 41598_2024_84965_MOESM5_ESM.pdf]

Title: Development of the Membrane Ceiling Method for in vitro Spermatogenesis

Authors:

Maki Kamoshita[1], Hiroki Shirai[2], Hiroko Nakamura[2], Tetsuya Kishimoto[2], Yuki Hatanaka[3], Daisuke Mashiko[3], Katsuhiro Esashika[4], Jingjing Yang[5], Satoshi Yamasaki[5], Takehiko Ogawa[6], Hiroshi Kimura[2*], Masahito Ikawa[1, 3, 7, 8*]

1 Research Institute for Microbial Diseases, Osaka University, Osaka, Japan.

2 Micro/Nano Technology Center, Tokai University, Kanagawa, Japan

3 Immunology Frontier Research Center, Osaka University, Osaka, Japan

4 Biotechnology Department, Synthetic Chemicals Laboratory, R&D Center, Mitsui Chemicals, Inc., Chiba, Japan

5 Marketing & Innovation Department, New Business Incubation Center, Mitsui Chemicals, Inc., Tokyo, Japan

6 Institute of Molecular Medicine and Life Science, Yokohama City University, Yokohama, Japan

7 The Institute of Medical Science, The University of Tokyo, Tokyo, Japan

8 Center for Infectious Disease Education and Research, Osaka University, Osaka, Japan

Supplementary Fig. S1

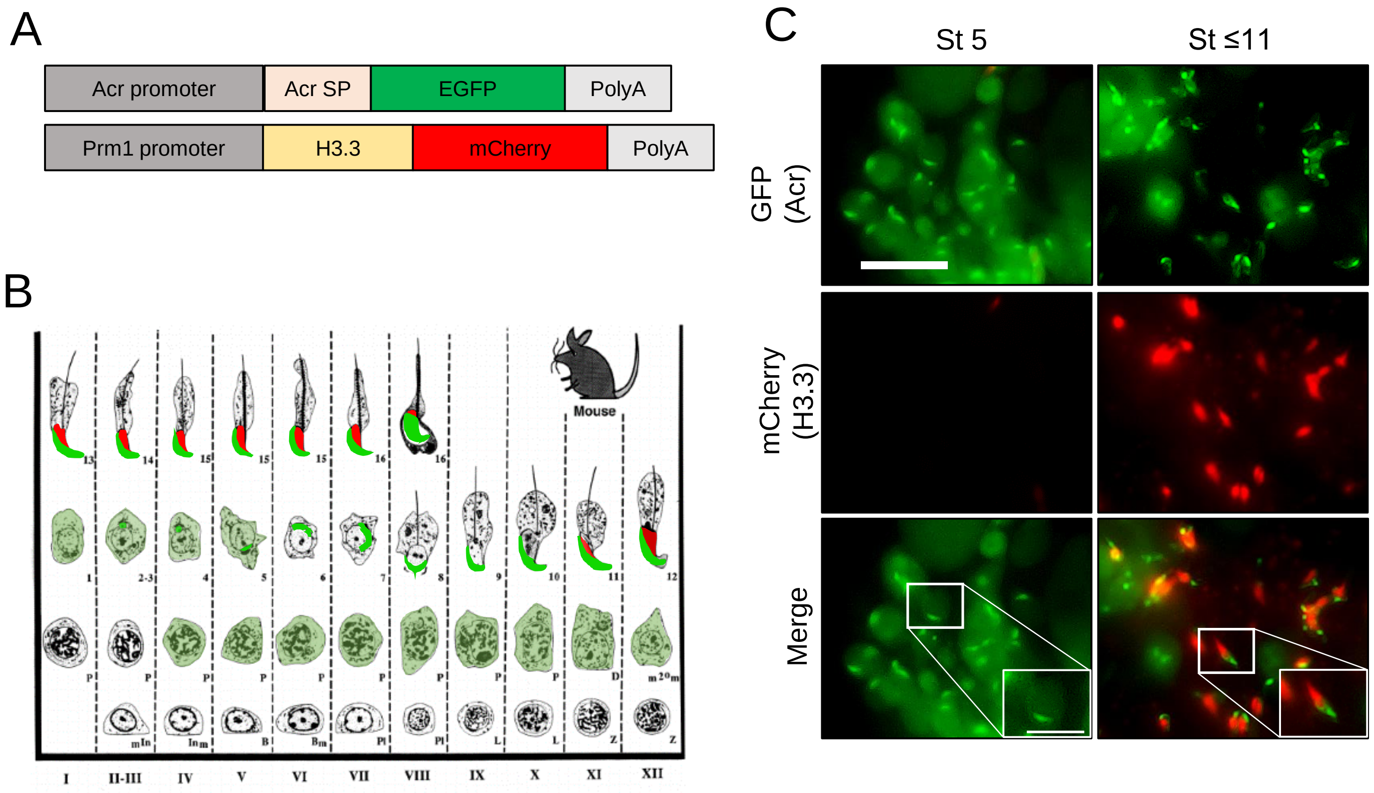

modified from Russell et al., 1990

Supplementary Fig. S1 Generating Green-Acr/Red-Nucleus (GARN) mice. (A) Constructs used for generating GARN mice. Acr3-EGFP (upper) and Prm1-H33-mCherry (lower). Prm1-H33-mCherry was gifted from Dr. Yuki Okada (Tokyo University). (B) A schematic image of the fluorescent protein localization during spermatogenesis in GARN mice. (C) Fluorescent signals of the step 5 (St5) and step ≤11 (St≤11) spermatids collected from adult GARN mice. Scale bar = 20 μm and 10 μm (zoom).

Supplementary Fig. S2

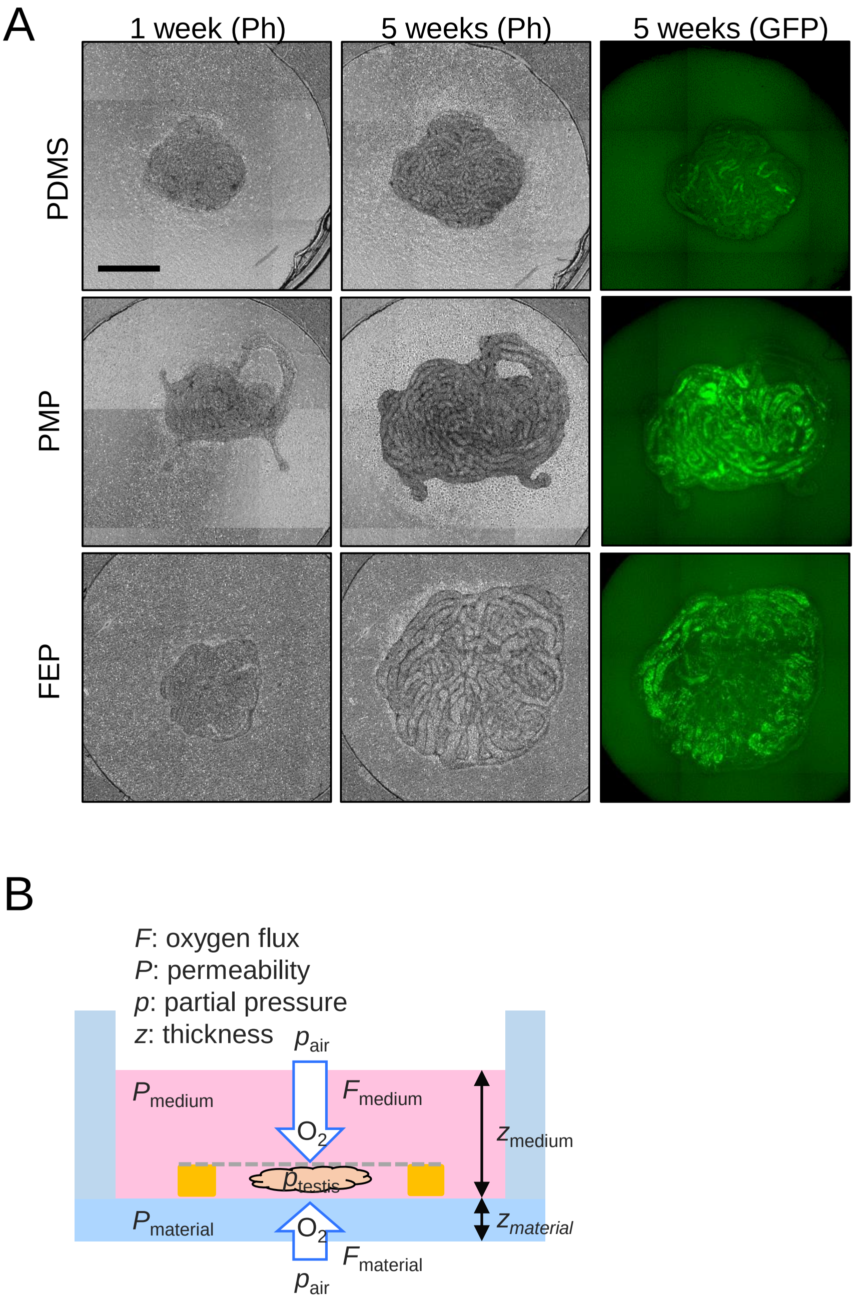

Supplementary Fig. S2 (A) Representative images of tissue expansion from 1 week to 5 weeks and GFP expressing area at 5 weeks cultured in the MC cihp with the PDMS, PMP, and FEP bottom well plate or dish. Ph; phase contrast. Scale bar = 1 mm. (B) A schematic image for calculation of maximum oxygen flux.

Supplementary Fig. S3

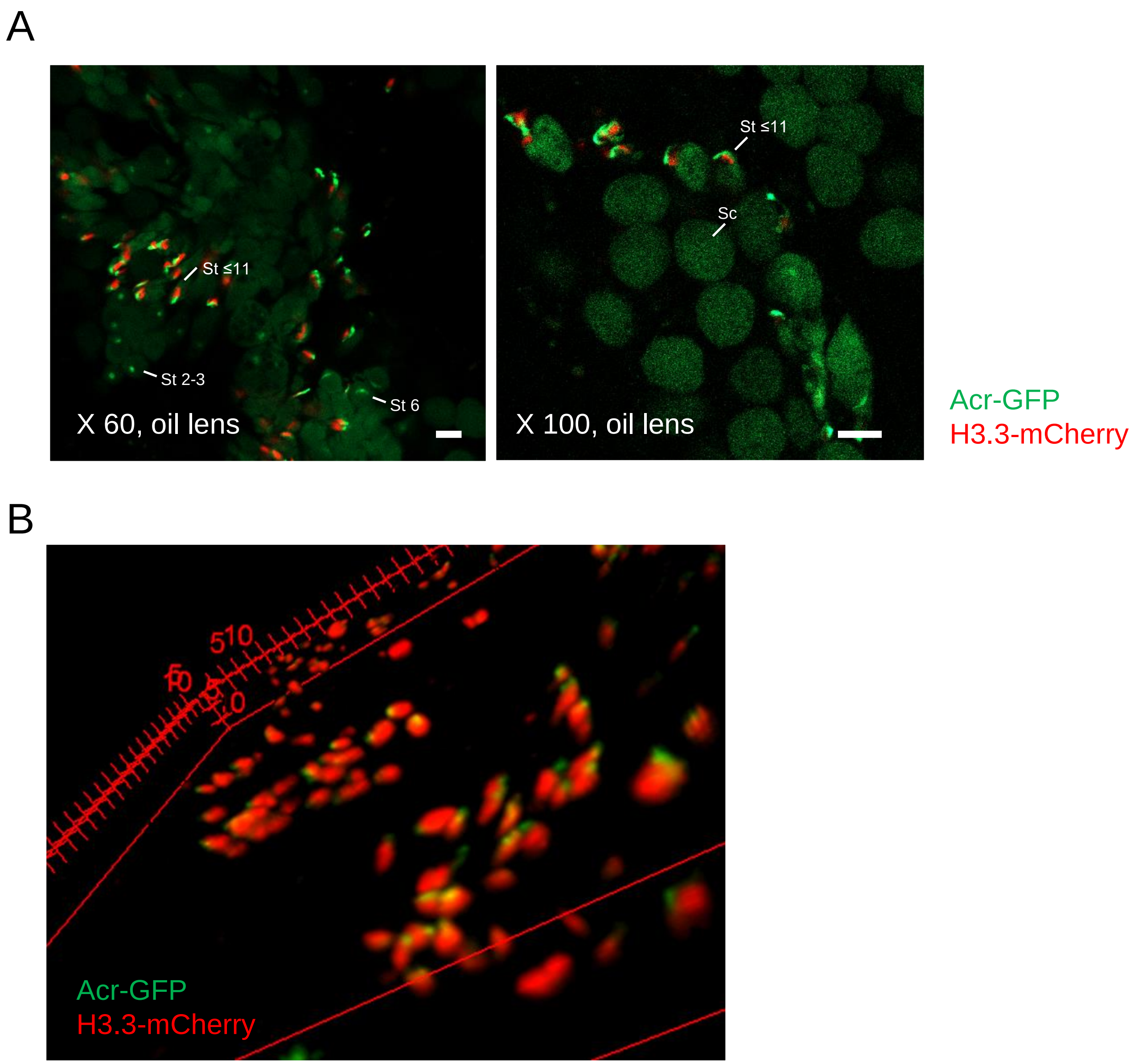

Supplementary Fig. S3 Representative images taken by a confocal microscope (A) Images were taken using (left) 60 times and (right) 100 times objective lens of a confocal microscope (Eclipse Ti). Scale bars = 10 µm. Sc; spermatocytes, St; spermatids (B) 3D image of sperm heads constructed from z-stack images.

Supplementary Table S1. Properties of microporous membranes

Material	Pore size (μm)	Porosity (%)	CAT No.
polycarbonate (PC)	0.4	18.8	1000M25/811N403[1]
	10.0	5-20	TCTP02500[2]
polyethylene terephthalate (PET)	0.45	0.6	2000M12/640N453[1]
	3	5.7	2000M12/580M303[1]

1. it4ip, Louvain-la-Neuve, Belgium, https://www.it4ip.be/wp-content/uploads/2023/03/PORTFOLIO-_-ipCELLCULTURE-PC-PET.pdf (Retrieved August 19, 2024)

2. Merck KGaA, Darmstadt, Germany, https://www.merckmillipore.com/JP/en/product/Isopore-Membrane-Filter,MM_NF-TCTP02500 (Retrieved August 19, 2024)

Supplementary Table S2 Properties for calculating oxygen permeability, and calculated maximum oxygen flux in medium and PDMS, PMP, and FEP plates.

	Medium	PDMS	PMP	FEP
Oxygen permeability (P) (pmol/cm·s·mmHg)	3.20×10^{-2} [3, 4]	43.5×10^{-2} [3,4]	1.25×10^{-2} [5]	0.142×10^{-2} [6]
Thickness (z) (cm)	0.2	5.0×10^{-2}	5.0×10^{-3}	2.5×10^{-3}
Partial pressure in air (p_{air}) (mmHg)	141.4 [7]	141.4 [7]	141.4 [7]	141.4 [7]
Partial pressure in tissue (p_{testis}) (mmHg)	0	0	0	0
Maximum oxygen flux (F) (pmol/cm²·s)	22.6	1253	376.1	102.9

3. L. Sønstevold, et al., Application of Polymethylpentene, an Oxygen Permeable Thermoplastic, for Long-Term on-a-Chip Cell Culture and Organ-on-a-Chip Devices, Micromachines, 14(3), 532, 2023

4. M. Nishikawa, et al., Stable Immobilization of Rat Hepatocytes as Hemispheroids On to Collagen-Conjugated Poly-Dimethylsiloxane (PDMS) Surfaces: Importance of Direct Oxygenation Through PDMS for Both Formation and Function, Biotechnology and Bioengineering,99(6), 1472-1481, 2008.

5. M. Nishikawa, et al., Accurate Evaluation of Hepatocyte Metabolisms on a Noble Oxygen-Permeable Material With Low Sorption Characteristics, Frontiers in Toxicology, 4, 810478, 2022.

6. Daryl. E. Powers, et al., Accurate control of oxygen level in cells during culture on silicone rubber membranes with application to stem cell differentiation, Biotechnology Progress, 26(3), 805-818, 2009.
Trenton. L. Place, et al., Limitations of oxygen delivery to cells in culture: An underappreciated problem in basic and translational research, Free Radical Biology and Medicine, 113, 311-322, 2017.

7. Trenton. L. Place, et al., Limitations of oxygen delivery to cells in culture: An underappreciated problem in basic and translational research, Free Radical Biology and Medicine, 113, 311-322, 2017.

Supplementary Movie legends

Supplementary Movie S1 Time lapse imaging of testis tissue in bright field. The image taken once per week for 14 weeks by BiostationCT during culture on PMP.

Supplementary Movie S2 Time lapse imaging of testis tissue in GFP. The image taken once per week for 14 weeks by BiostationCT during culture on PMP. Green: Acr-GFP.

Supplementary Movie S3 Time lapse imaging of spermatogenesis at the same point of the seminiferous tubule. The image taken once per day for 12 days by BiostationCT during culture on PMP. Green: Acr-GFP. Red: H3.3-mCherry.

Supplementary Movie S4 3D imaging of organelle formation of spermatids by the confocal microscope. Green: Acr-GFP. Red: H3.3-mCherry.
